# Common writings of baby names in Japan, 1989–2003: Explanation of survey data

**DOI:** 10.1016/j.dib.2021.107678

**Published:** 2021-12-04

**Authors:** Yuji Ogihara

**Affiliations:** Institute of Arts and Sciences, Tokyo University of Science, Kagurazaka, Shinjuku-ku, Tokyo, 162-8601, Japan

**Keywords:** Name, Baby, Writing, Chinese character, Hiragana, Survey, Sample size, Japan

## Abstract

Previous research on Japanese names has analyzed the surveys on baby names conducted by Meiji Yasuda Life Insurance Company. The company displayed the yearly top 10 common writings of baby names between 1989 and 2003. However, it was unclear how the surveys for those 15 years were conducted. The data are necessary to evaluate the usableness of the surveys and conduct empirical research. Therefore, I asked the company for further data about the surveys. The methods of the surveys were consistent not only between 1989 and 2003, but also consistent with those between 2004 and 2018. The analyses on the annual sample sizes by gender showed that the surveys between 1989 and 2003 are comparable to the surveys between 2004 and 2018. The company is unable to access the raw data of these surveys, which makes it impossible to provide results other than the top 10 most common writings.

## Specifications Table

**Table:** 

Subject	Social Sciences
Specific subject area	Linguistics, Psychology, Sociology, Anthropology, Humanities, Arts, Decision Sciences, Data Science
Type of data	Table, Figure
How the data were acquired	Survey
Data format	Raw
Description of data collection	Meiji Yasuda Life Insurance Company conducted surveys on baby names every year between 1989 and 2003. They collected name data from their policyholders.
Data source location	Institution: Meiji Yasuda Life Insurance Company, City: Tokyo, Country: Japan
Data accessibility	Repository name: Open Science Framework (OSF) Data identification number: 10.17605/OSF.IO/ZA6X7 Direct link to the dataset: https://doi.org/10.17605/OSF.IO/ZA6X7 Table S1. Sample sizes of the surveys, 1989-2018. Table S2. Top 10 most common writings of baby names, 1989-2018.

## Value of the Data

• In Japan, sizable and reliable name data are scarce unlike in the United States (e.g., [1,2]) and China (e.g., [3,4]), which has prevented empirical research on names in Japan [5]. Thus, the data are valuable to advance empirical research on names.
• Those who want to do empirical research on names in Japan can use/benefit from the data.
• The data can be used for further insights/analyses on names in Japan (e.g., historical changes in names, cultural similarities/differences in names).

## 1 Data Description

Researchers have investigated names as an important element of language. For instance, Ogihara [6] analyzed recent Japanese baby names and demonstrated that even when the same Chinese characters are used for names, there are many readings of them (e.g., a common boys’ writing of name “大翔” had 18 variations in readings). This study provides empirical evidence of the difficulty in reading recent Japanese names correctly, showing that there are many readings in Chinese characters used in Japan (particularly when used for human names). In addition, Ogihara [7,8] systematically categorized uncommon names in recent Japan and explained how they are read, which contributes to a better understanding of the Japanese language.

Moreover, names have been analyzed to empirically examine psychology, behavior, and culture. For example, Ogihara et al. [9] indicated that the rates of common names decreased in Japan between 2004 and 2013. Further, Ogihara [10] showed that the rates of unique names increased in Japan between 2004 and 2018. These studies suggest that Japanese culture increasingly emphasized uniqueness-seeking and became more individualistic over time (e.g., [11], [12], [13], [14]; for reviews see, [15,16]).

In these studies, data from a private company in Japan, Meiji Yasuda Life Insurance Company, have played an important role. Meiji Yasuda has conducted surveys of baby names every year for more than 30 years from 1989 to the present [17]. Meiji Yasuda has publicly introduced their name surveys and displayed some results on their website every year from 2004 to the present. Because baby names attract much attention from people, the company highly announces their annual survey and its results at the end of the year.

However, Meiji Yasuda has not sufficiently explained their surveys which were conducted between 1989 and 2003. Although after 2004, the company has made their original featured web page every year where they have published their surveys, they have not done so for the surveys between 1989 and 2003. Additionally, while the company has shown various results of the surveys after 2004 (e.g., the top 100 most common writings of baby names, the top 50 most common readings, the lists of readings of the top 10 most common writings), the company shows only the top 10 most common writings of baby names before 2004. Further, regarding the method they used, they only mention that they conducted name surveys on policyholders of their insurances. Thus, it is unclear how the surveys between 1989 and 2003 were conducted and to what extent their results are reliable.

In Japan, there are no systematic name databases that the government or its official organization deals with and that are available to researchers [5], unlike in the United States (e.g., [1,2]) and China (e.g., [3,4]). Thus, reliable and sizable name data in Japan are valuable. In particular, name data in older times are precious because they are scarcer and tracing back into the past is generally difficult in research. Analyzing these data can contribute to a deeper and broader understanding of the phenomena regarding many objects such as language, psychology, behavior, and culture.

To overcome this limitation, I asked the company for further information of their surveys and obtained some valuable data.^1^ These data are not provided on their website at least at present. Here, I provide the new data, analyze them, and summarize the surveys for following future research.

The data are available online (https://doi.org/10.17605/OSF.IO/ZA6X7). Table S1 summarizes the sample sizes of the surveys between 1989 and 2018. Table S2 summarizes the top 10 most common writings of baby names between 1989 and 2018.

## 2 Experimental Design, Materials and Methods

### 2.1 Methods of the surveys

The website writes that they have conducted surveys on their new policyholders and the stored information of their known policyholders every year since 1989 [17].

However, there is no detailed explanation of the methods they used. Thus, I asked the company about them. It was revealed that the methods of the surveys were consistent between 1989 and 2003. Further, it was confirmed that the methods of the annual surveys conducted between 1989 and 2003 were same as those of the annual surveys conducted after 2004 [9,17]. Therefore, it is possible to compare the surveys within the 15 years between 1989 and 2003 and compare them with the surveys after 2004.

### 2.2 Sample sizes of the surveys

**Fig. 1:**
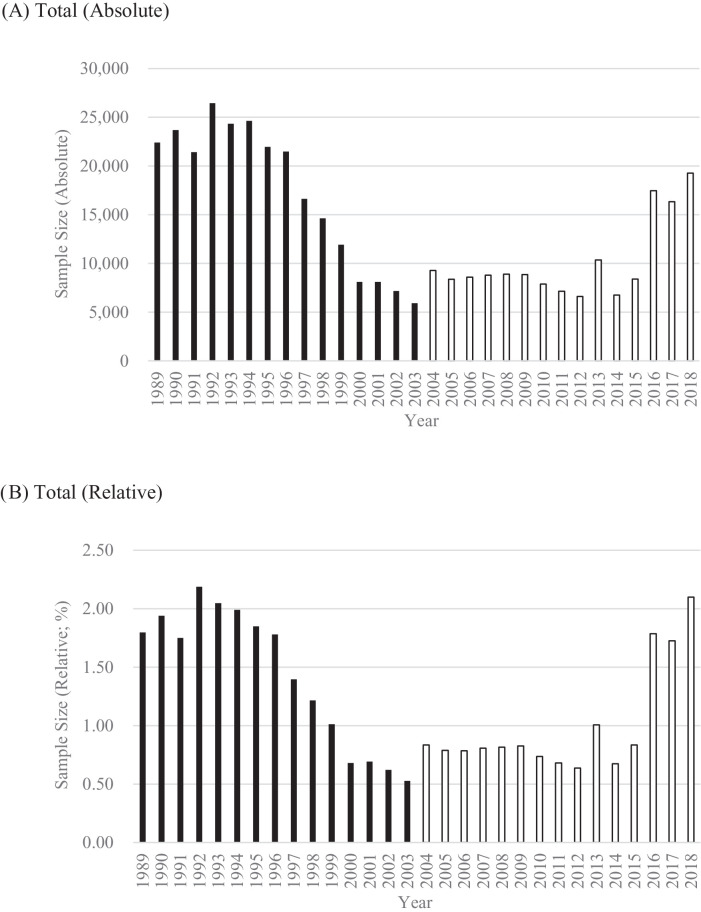

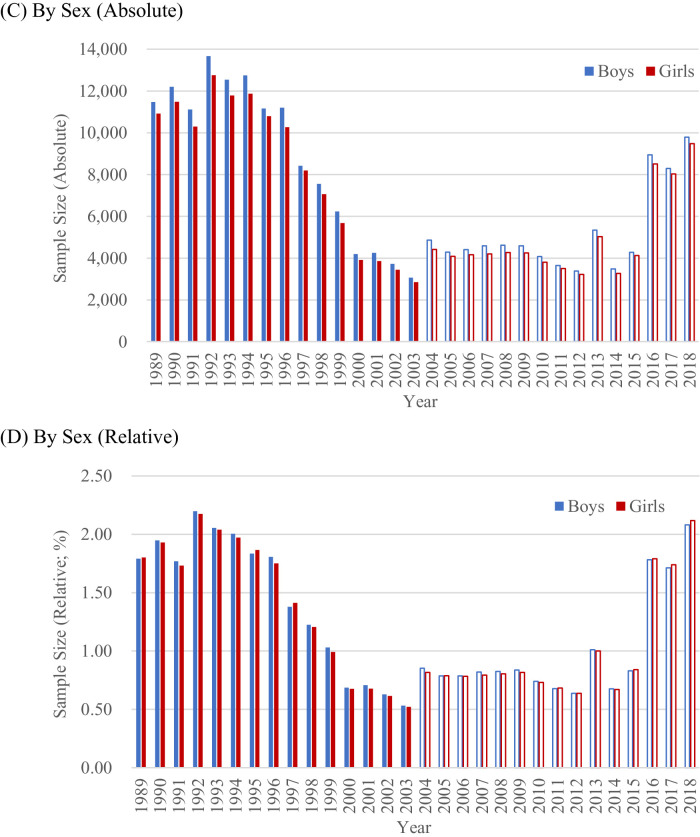
Sample sizes of the surveys, 1989-2018.

Note. Relative sample sizes were calculated by dividing absolute sample sizes by numbers of births. To facilitate the understanding of the surveys between 1989 and 2003, the results of the surveys between 2004 and 2018 are also shown.

The sample sizes are summarized in Table S1 and Fig. 1. The total sample size was 258,807 (Boys: 133,597, Girls: 125,210), and the annual average of the total sample size was 17,254 (Boys: 8906, Girls: 8347). These are larger than the total sample size (152,995; Boys, 78,623; Girls, 74,372) and the annual average of the total sample size (10,200; Boys, 5242; Girls, 4958) of the surveys for the next 15 years between 2004 and 2018.

The annual sample sizes ranged from 5921 to 26,438. They were over 20,000 from 1989 to 1996, but they gradually decreased from 1997 to 2003. Nevertheless, the sample size in 2003 was over 5000 (5921). These temporal trends within 1989-2003 and differences in the surveys between 1989-2003 and 2004-2018 were consistent for boys and girls.

**Fig. 2:**
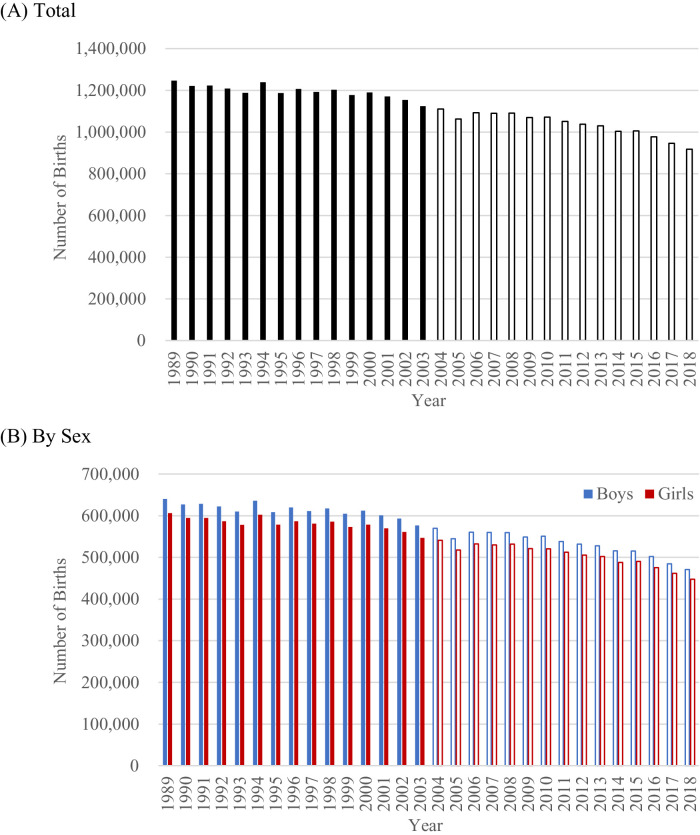
Number of births in Japan, 1989-2018.

I also examined how each annual sample size was relatively large. Specifically, I calculated the percentages of the sample sizes out of the sizes of population of interest (the number of babies who were born in a given year). Because the number of births gradually decreased in Japan for this period (Fig. 2; [18]), the relative sample size may be affected by this decrease in the population of interest.

Despite the decrease in population, the pattern of the relative sample sizes was same as that of the absolute sample sizes. The relative sample sizes between 1989 and 2003 were larger than those between 2004 and 2018. Further, these historical trends within 1989-2003 and the differences in the surveys between 1989-2003 and 2004-2018 were consistent for boys and girls. These analyses show that the surveys between 1989 and 2003 are comparable to the surveys after 2004.

### 2.3 Results of the surveys

The company displayed the rankings of the top 10 most common writings (Table S2; [17]). In Japan, three types of characters can be used for names: Chinese characters, hiragana, and katakana. In these rankings, all boys’ names and almost all girls’ names consisted of Chinese characters. Some girls’ names were written in hiragana, with only two variations, Sakura (さくら; meaning a cherry blossom/tree) and Hinata (ひなた; meaning a sunny place). Names written in katakana were not included in the top 10 common writings between 1989 and 2003.

The numbers of names at the same rank due to the same number of names were also counted. Because of these names at the same rank, the number of variations in the top 10 most common writings can include more than 10 variations. For example, there were three girls’ names in the 10th rank in 1999 (彩乃, 優花, and 葵), leading to 12 variations in the top 10 most common ranking. These should be moderated in analyses.

I asked the company whether they could provide results other than the top 10 most common writings. For example, the number of babies in each rank is valuable information. Moreover, readers may be interested in not only common writings but also common readings because there are many readings for a writing [6]. Indeed, these results have been shown after 2004 to the present [9,17]. They replied that they were unable to access the raw data and thus they could not conduct additional analyses anymore and display the results. This is unfortunate, but it is also important to know that there is no other information available, which is different from the state where there is other information available but it is not openly displayed.

### 2.4 Summary

To summarize, new data that I obtained from Meiji Yasuda include 1) information that the methods of the surveys were consistent between 1989 and 2003 and same as those of the surveys conducted after 2004, 2) annual sample sizes by gender, and 3) information that they were unable to access the raw data and thus they could not conduct additional analyses.

I conducted analyses on the annual sample sizes by gender and showed that the surveys between 1989 and 2003 are comparable to the surveys after 2004. Furthermore, I added some information about the top 10 most common writings of baby names between 1989 and 2003: the numbers of variations, same-rank names, hiragana names, and katakana names.

In Japan, sizable and reliable name data are scarce unlike in the United States (e.g., [1,2]) and China (e.g., [3,4]). Therefore, these new data are valuable to advance empirical research on names in Japan. The data can be used for further insights/analyses on names in Japan (e.g., historical changes in names, cultural similarities/differences in names).

## CRediT Author Statement

**Yuji Ogihara:** Conceptualization, Methodology, Validation, Formal analysis, Investigation, Data curation, Writing - original draft, Writing - review & editing, Visualization, Supervision, Project administration.

## Declaration of Competing Interest

The authors declare that they have no known competing financial interests or personal relationships that could have appeared to influence the work reported in this paper.

## Footnotes

1 I also obtained permission from Meiji Yasuda to show their information in academic articles.
